# Changes in smoking behaviour among healthcare workers with COVID-19

**DOI:** 10.1192/j.eurpsy.2023.1413

**Published:** 2023-07-19

**Authors:** H. Ziedi, M. Mersni, D. Brahim, G. Bahri, N. Mechergui, I. Youssef, S. Ernez, H. Ben Said, N. Ladhari

**Affiliations:** 1 Charles Nicolle hospital, Tunis, Tunisia

## Abstract

**Introduction:**

The COVID-19 pandemic had a considerable psychosocial impact on healthcare workers (HCWs) who were constantly requested during this era with an increased risk of infection. This implies behavioural changes, especially in smoking behaviour.

**Objectives:**

To study smoking behaviour in HCWs with COVID-19.

**Methods:**

A cross-sectional descriptive study conducted in the department of occupational pathology of Charles Nicolle Hospital in Tunis involving the smoking HCWs affected by COVID-19 during the period from September 1, 2020, to February 28, 2021. The data collection was carried out by a telephone call using a standardized questionnaire.

**Results:**

During the study period, 61 smoking HCWs were identified. Thirty-two patients agreed to answer the questionnaire, with a response rate of 52%. The mean age was 41±10 years. The sex ratio (M/F) was 1.46. Half of the participants had comorbidities. The most represented occupational categories were blue-collar workers (n=11) followed by nurses (n=10) and physicians (n=7). The median professional seniority was 13 [3.5; 20] years. The mean age of smoking initiation was 20±5 years. The most common mode of smoking was cigarettes (93%) with an average consumption of 19 cigarettes per day. Water pipe smoking was noted in 3 patients. All patients started smoking before the COVID-19 infection. Strong tobacco dependence was noted in 25% of patients. Twenty-one per cent of the population had moderate dependence. Half of the participants maintained the same level of smoking as before the COVID-19 infection. An increase in smoking was noted in 34% of patients. A decrease in the level of smoking was reported by 15% of respondents. Four participants stopped smoking after COVID-19 infection. The reasons for smoking cessation were COVID-19 damage (n=3) and confinement with family (n=1).

**Conclusions:**

The change in smoking behaviour during the COVID-19 pandemic is notable, particularly in HCWs who are exposed to a high physical and mental load. The presumed association of smoking with severe forms of COVID-19 infection makes tobacco control in HCWs an obligation in order to preserve the continuity of care.

**Disclosure of Interest:**

None Declared

